# Mechanistic target of rapamycin complex 1 orchestrates the interplay between hepatocytes and Kupffer cells to determine the outcome of immune-mediated hepatitis

**DOI:** 10.1038/s41419-022-05487-0

**Published:** 2022-12-09

**Authors:** Xiaoli Sun, Yajie Ni, Qingmiao Lu, Yan Liang, Mengru Gu, Xian Xue, Chunsun Dai

**Affiliations:** 1 Department of Clinical Genetics, 2nd Affiliated Hospital, Nanjing Medical University, Nanjing, Jiangsu China; 2 Center for Kidney Disease, 2nd Affiliated Hospital, Nanjing Medical University, Nanjing, Jiangsu China

**Keywords:** Apoptosis, Autoimmunity, Autoimmune hepatitis

## Abstract

The cell-cell interaction between hepatocytes and Kupffer cells (KCs) is crucial for maintaining liver homeostasis, and the loss of KCs and hepatocytes is known to represent a common pathogenic phenomenon in autoimmune hepatitis. Until now, the mechanisms of cell-cell interaction between hepatocytes and KCs involved in immune-mediated hepatitis remains unclear. Here we dissected the impact of activated mTORC1 on the cell-cell interaction of KCs and hepatocyte in immune-mediated hepatitis. In the liver from patients with AIH and mice administrated with Con-A, mTORC1 was activated in both KCs and hepatocytes. The activated mTORC1 signal in hepatocytes with Con-A challenge caused a markedly production of miR-329-3p. Upregulated miR-329-3p inhibited SGMS1 expression in KCs through paracrine, resulting in the death of KCs. Most of maintained KCs were p-S6 positive and distributed in hepatocyte mTORC1 negative area. The activation of mTORC1 enabled KCs expressed complement factor B (CFB) to enhance the complement alternative system, which produced more complement factors to aggravate liver injury. Our findings remonstrate a heterogeneous role of mTORC1 in specific cell type for maintaining tolerogenic liver environment, and will form the basis for the development of new interventions against immune-mediated hepatitis.

## Introduction

The liver is a central metabolic organ that is constantly exposed to gut-derived nutrients and to antigens from aged or damaged cells. In homeostasis, the “liver tolerance effect” mediates local and systemic tolerance to self and foreign antigens, and the non-parenchymal cells responsible for the liver tolerance are resident dendritic cells, sinusoidal endothelial cells, Kupffer cells (KCs), and hepatic stellate cells [1]. Conversely, hepatocytes represent the parenchymal cell population in the liver, and these cells mainly perform metabolic functions. However, hepatocytes also participate in immune regulation by directly acting as the primary sensors and triggers of the immune responses induced by antigens and drugs [2].

KCs, the resident macrophages in the liver, originate from fetal liver-derived erythromyeloid progenitor cells expressing macrophage colony-stimulating factor-1 receptor [3]. The interaction of KCs and hepatocytes in the space of Disse is functionally important: During homeostasis, KCs can either exert a negative regulatory effect on hepcidin mRNA expression in hepatocytes [4], or deliver the iron that KCs acquire from dead red blood cells to hepatocytes [5]. Moreover, the KC niche is composed by the coordinated interactions with hepatocytes in which ID3 expression is induced and endothelial cells and stellate cells in which LXR-a expression is induced [6]. These findings indicate that the cell-cell interaction between hepatocytes and KCs is critical for maintaining liver homeostasis.

During acute liver failure, initial hepatocyte injury is mediated by oxidative stress, and then the damage-associated molecular patterns released from damaged hepatocytes activate KCs [7]. The major sources of reactive oxygen species in acute liver failure include KCs, neutrophils, and dysfunctional mitochondria [8], and, accordingly, KC depletion alleviates acute liver injury induced by ischemia/reperfusion (I/R) [9], concanavalin A (Con-A) [10], or lipopolysaccharide [11]. Borst et al. found that KCs are rapidly lost and then replaced by infiltrating blood monocytes in the course of viral hepatitis [12], and a similar phenomenon was observed in hepatic inflammation induced by acetaminophen, CCl_4_, or bacterial infection [13–15]. In all these studies, a temporary decline of KC numbers was observed within 24 h, which suggests that KC loss is a common event during acute hepatitis. However, the role and mechanism of action of hepatocytes in KC loss in acute hepatitis remain unclear.

Mammalian target of rapamycin (mTOR) plays a vital role in regulating mRNA translation, metabolism, and protein turnover [16], and mTOR complex 1 (mTORC1) signaling has been widely reported to be involved in acute hepatic I/R, cisplatin induced-acute kidney injury and postnatal brain development [17–19]. In this study, we found that the content of p-S6 in all hepatocytes was very high at 1 h after Con-A challenge, and the p-S6-positive hepatocytes presented a plaque-like distribution after 3 h; more interestingly, most of the remaining KCs were p-S6 positive and were distributed in regions that were negative for p-S6-enriched hepatocytes. Thus, the mTORC1-activated KCs and hepatocytes appear to localize in a mutually exclusive manner.

Here, we sought to investigate the role and mechanisms of mTORC1 activation in hepatocytes and KCs in the initiation of hepatitis. By using hepatocyte-specific or macrophage-specific *Tsc1*-knockout mice, we found that mTORC1-activated hepatocytes protect the liver from Con-A-induced hepatitis by killing the surrounding KCs, whereas mTORC1-activated KCs aggravate liver injury by stimulating the complement alternative system.

## Results

### Activation of mTORC1 in hepatocytes and KCs of patients with AIH and of mice with immune-mediated hepatitis

**Fig. 1:**
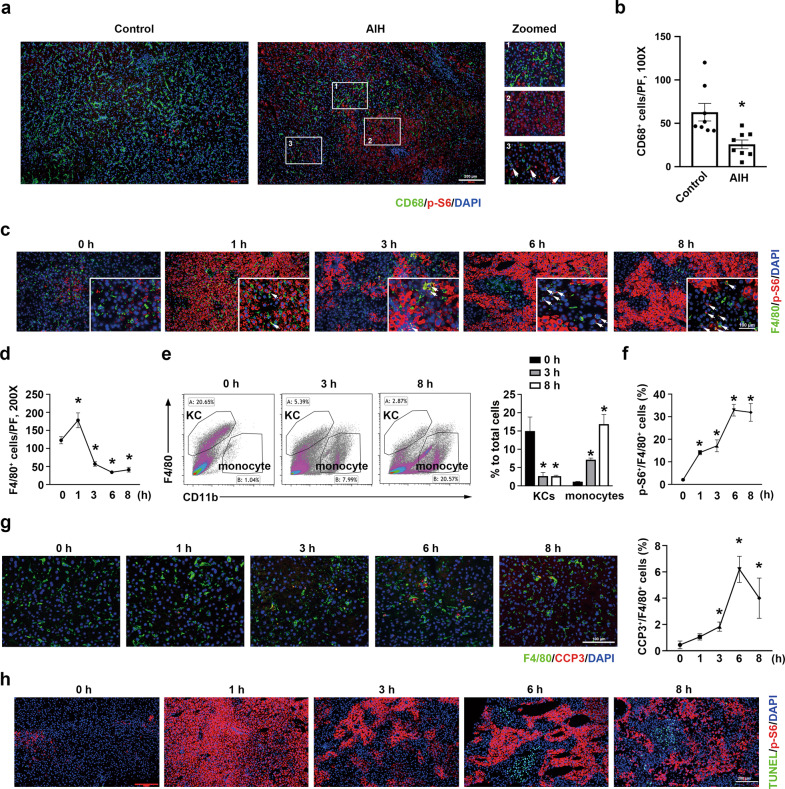
KC loss and elevated mTORC1 expression in human and mouse inflammatory liver injury. **a** Immunohistochemical staining of CD68 (green) and p-S6 (red) in liver cross sections from control and patients with autoimmune hepatitis (AIH) (box point towards enlarged pictures at the right side). Scale bar = 200 μm. **b** Quantitative determination of CD68^+^ cells among groups as indicated, control = 8, AIH = 8. **c** Representative co-immunofluorescent staining images for F4/80 with p-S6 (white arrows). Scale bar = 100 μm. **d** Quantitative determination of F4/80^+^ cells among groups as indicated, *n* = 3. **e** Flow cytometry analysis of KCs. Each box indicates bone marrow ­derived monocytes, CD11b^+^F4/80^low^; KCs, CD11b^low^F4/80^+^. Right, mean percentage of KCs and monocytes, *n* = 4. **f** Quantitative determination of F4/80^+^ and p-S6^+^ cells among groups as indicated, *n* = 3. **g** Left, representative co-immunofluorescent staining images for F4/80 with cleaved caspase 3 (CCP3). Scale bar = 100 μm. Right, quantitative determination of CCP3^+^ and F4/80^+^ cells among groups as indicated. *p < 0.05 versus control mice, *n* = 3. **h** Co-immunofluorescent stained with TUNEL and p-S6. Scale bar = 200 μm.

We initially stained liver biopsies from AIH patients with CD68 and p-S6. In liver samples from patients with AIH, CD68 staining showed a markedly loss of KCs (Fig. 1a, b). A similar pattern of KC loss was observed in livers derived from mice subjected to Con-A treatment, a well-established experimental model of immune-mediated hepatitis (Fig. 1c–e, Supplementary Fig. 1a, b). KC death was increased from 3 h after Con-A injection (Fig. 1d, g). These findings indicated that immune hepatitis in both humans and mice shows characteristic features of KC loss. p-S6 staining, one of major downstream molecules of mTORC1, partial enhanced hepatocyte and KC expression in liver biopsies from patients with AIH was observed (Fig. 1a). Similarly, western blotting and immunostaining showed that Con-A-induced hepatitis was associated with elevated levels of p-S6 protein in liver tissues of mice (Fig. 1c, f, Supplementary Fig. 1c).

Interestingly, the distribution of KCs in livers from immune-mediated hepatitis was not uniformly. The immunostaining staining showed the KCs remained predominantly in the focal areas of weak p-S6 staining in the liver from AIH patients and Con-A injected mice (Fig. 1a, c). Intriguingly, the percentage of KCs showing mTORC1 activation was elevated during the observation period (Fig. 1f). Co-staining of p-S6 and TUNEL revealed that at 6 and 8 h after Con-A injection, the liver displayed focal areas of weak p-S6 staining coupled with large numbers of apoptotic cells, which suggested enhanced hepatocyte death (Fig. 1g). Collectively, our data indicate that the activation of mTORC1 in hepatocytes leads to the loss of KCs, and the hepatocyte injury is associated to the p-S6 positive KCs.

### Activation of mTORC1 in hepatocytes promotes KCs loss and protects against Con-A induced hepatitis in mice

**Fig. 2:**
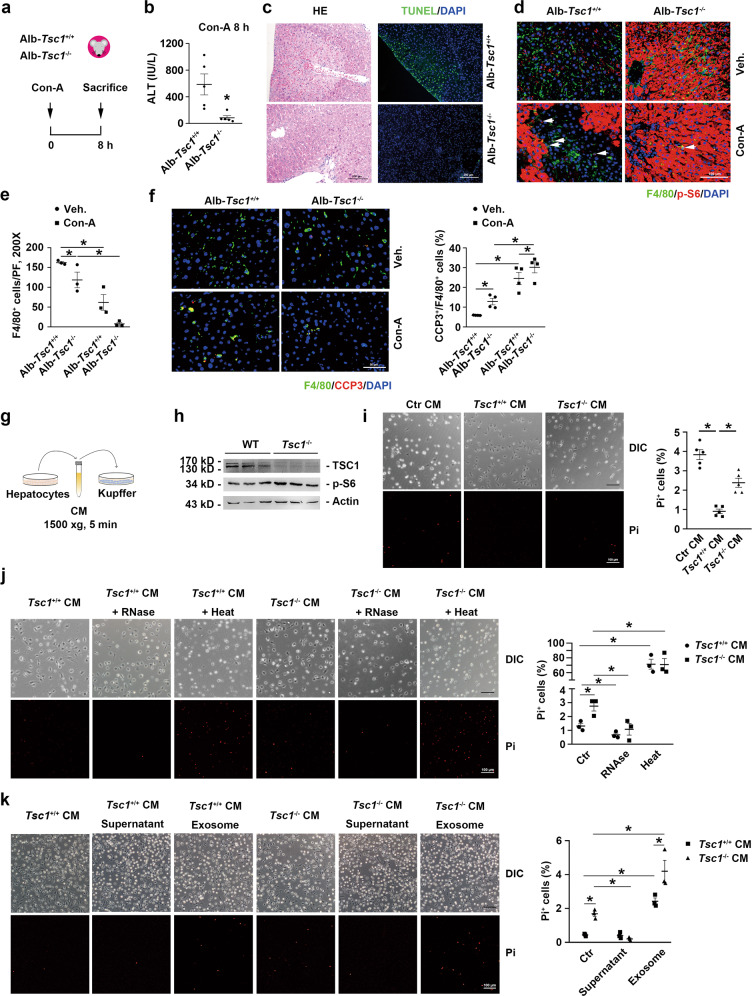
Hepatocytes *Tsc1* ablation protects liver against Con-A induced hepatocyte injury. **a** The strategy for hepatocytes *Tsc1* deletion in Alb-Cre^+/−^, *Tsc1*^fl/fl^ mice with Con-A administration. **b** The ALT levels in serum, *n* = 6. **c** Representative HE and TUNEL- stained mouse livers. Scale bar = 200 μm. **d** Representative co-immunofluorescent staining images for F4/80 with p-S6 (white arrows). Scale bar = 100 μm. **e** Quantitative determination of F4/80^+^ cells among groups as indicated, *n* = 3. **f** Left, representative co-immunofluorescent staining images for F4/80 with CCP3 (white arrows). Scale bar = 50 μm. Right, quantification, *n* = 4. **g** The co-culture system. **h** Western blot assay showing the abundance of TSC1 in primary hepatocytes. **i** Left, representative pi-staining images for primary KCs were cultured with conditioned media (CM) from *Tsc1*^-/-^ hepatocytes for 48 h. Scale bar = 100 μm. Right, quantitative determination of Pi^+^ KCs among groups as indicated, *n* = 5. **j** Left, representative Pi-staining images for primary KCs cultured with CM treated with RNase or 99 °C boiled for 48 h. Scale bar = 100 μm. Right, quantitative determination of Pi^+^ KCs among groups as indicated, *n* = 3. **k** Left, representative Pi-staining images for primary KCs cultured with exosomes or exosomes free-CM for 48 h, respectively. Scale bar = 100 μm. Right, quantification, *n* = 3. ^*^p < 0.05.

To evaluate the role of hepatocyte mTORC1 activation in KC maintenance and hepatitis, we generated a mouse model with hepatocyte-specific *Tsc1* ablation (Alb-*Tsc1*^-/-^ mice; Supplementary Fig. 2a). When we injected Con-A into the hepatocyte-deficient mice and control littermates (Fig. 2a), we found that the Alb-*Tsc1*^-/-^ mice were largely protected against Con-A-induced liver injury, as indicated by diminished plasma ALT levels (Fig. 2b), a smaller necrotic area, and decreased hepatocyte apoptosis relative to the corresponding levels in Alb-*Tsc1*^+/+^ mice (Fig. 2c). Moreover, KC numbers were decreased and KC apoptosis was increased in the Alb-*Tsc1*^-/-^ liver as compared with the levels in the Alb-*Tsc1*^+/+^ liver with or without Con-A injection (Fig. 2d–f), which suggested that mTORC1 activation in hepatocytes might promote KC death. Furthermore, at 8 h after Con-A injection, T-cell and neutrophil infiltration were similar in the liver in Alb-*Tsc1*^-/-^ and Alb-*Tsc1*^+/+^ mice (Supplementary Fig. 2c, d); by contrast, CD11b^+^ monocyte infiltration was diminished in the Alb-*Tsc1*^-/-^ liver relative to the levels in the Alb-*Tsc1*^+/+^ liver (Supplementary Fig. 2e).

Collectively, our results support the conclusion that specific activation of mTORC1 in hepatocytes might exacerbate KC loss and protects mice from Con-A-induced liver injury downstream of inflammatory cells infiltration and activation.

### mTORC1-activated hepatocytes promote KC death by secreting miR-329-3p and thereby downregulating sphingomyelin synthase 1 (SGMS1) expression in KCs

To study the mechanism by which mTORC1-activated hepatocytes promote KC loss, we cultured KCs with the conditioned medium (CM) harvested from cultured hepatocytes (Fig. 2g). Primary cultured hepatocytes from *Tsc1*^fl/fl^ mice were infected for 48 h with an adenovirus carrying Cre recombinase to induce target-gene ablation (Fig. 2h). Interestingly, the death of KCs cultured with *Tsc1*^+/+^ hepatocyte-derived CM was decreased as compared with that of KCs cultured with control medium, suggesting that hepatocytes provide certain pro-survival factors for KCs under physiological conditions. However, KCs cultured with *Tsc1*^-/-^ hepatocyte-derived CM displayed higher mortality than did KCs cultured with *Tsc1*^+/+^ hepatocyte-derived CM (Fig. 2i). Raptor is an adapter protein and kinase regulator for mTORC1 and deficiency of which results in reduced mTORC1 activity [20]. The expression of Raptor had no significant differences in livers from mice with Con-A treatment (Supplementary Fig. 3a). Then, siRNAs were transfected into primary hepatocytes to knock down *Raptor* expression (Supplementary Fig. 3b). The CM from *Raptor*-knocked down hepatocytes attenuated the death of KCs (Supplementary Fig. 3c). These data suggest that the activation of mTORC1 in hepatocytes promotes the KC depletion.

mTORC1 activation in hepatocytes might promote KC death by producing either increased amounts of certain pro-death substances and/or diminished amounts of pro-survival substances for KCs. To identify the substances from hepatocyte that regulate KC survival, the hepatocyte-derived CM was subject to RNase treatment and heating to deactivate RNAs and proteins, respectively. Increased cell death was detected in the case of KCs cultured with heat-pretreated CM from *Tsc1*^+/+^ hepatocytes, which suggested that hepatocytes produce certain proteins that maintain KC survival (Fig. 2j); however, cell death was similar in KCs cultured with heat-pretreated CM from *Tsc1*^+/+^ hepatocytes and *Tsc1*^-/-^ hepatocytes. Conversely, cell death was largely attenuated in KCs cultured with RNase-pretreated CM from *Tsc1*^-/-^ hepatocytes, which suggested that Tsc1^-/-^ hepatocytes produce certain RNAs that promote KC death (Fig. 2j). The exosomes from the CM of both *Tsc1*^+/+^ and *Tsc1*^-/-^ hepatocytes were isolated to incubating KCs. The results showed that the exosomes but not the supernatants from *Tsc1*^-/-^ hepatocytes markedly enhanced KC death, which indicated that exosome-enveloped RNAs mediate the effect of mTORC1-activated hepatocytes on KC death (Fig. 2k).

**Fig. 3:**
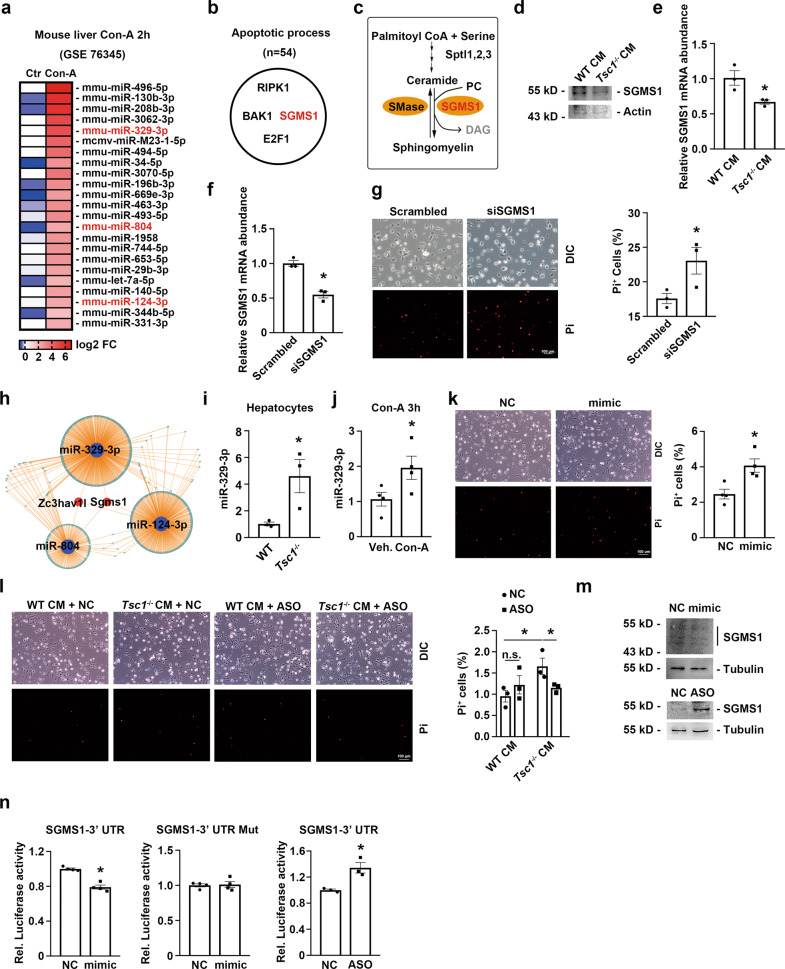
The death of KCs is induced by the reduction of SGMS1 which is targeted by miR-329-3p from mTORC1 activated hepatocytes. **a** Expression heatmap of the significantly upregulated miRNAs in mice injected with Con-A for 2 h as compared with normal controls (GSE76345) (fold change >4, *p* < 0.05, *n* = 4). **b** Gene ontology analysis of putative target genes of upregulated miRNAs using one selected gene ontology categories. **c** A scheme illustrating the role of SGMS1 which regulates the biotransformation of ceramide and sphingomyelin. Unmeasured analytes are shown in gray. **d**, **e** The mRNA and protein abundance of SGMS1 in primary KCs cultured with *Tsc1*^-/-^ primary hepatocytes CM for 48 h, *n* = 3. **f** the mRNA abundance of SGMS1 in KCs transfected with siRNA for SGMS1 (siSGMS1), *n* = 3. **g** Left, representative Pi-staining images for primary KCs transfected with siSGMS1 for 48 h. Scale bar = 100 μm. Right, quantitative determination of Pi^+^ KCs among groups as indicated, *n* = 3. **h** The miRNA-mRNA regulatory network. miRNAs were indicated to deformed blue, and mRNAs were indicated to deformed green or red, respectively. **i** miRNA abundance for miR-329-3p in *Tsc1*^-/-^ primary hepatocytes, *n* = 3. **j** miRNA abundance for miR-329-3p in liver tissues exposed to Con-A for 3 h, *n* = 4. **k** Left, representative Pi-staining images for primary KCs transfected with miR-329-3p mimic for 48 h. Scale bar = 100 μm. Right, quantitative determination of Pi^+^ KCs among groups as indicated, *n* = 4. **l** Left, representative Pi-staining images for primary KCs cultured with CMs for 48 h. The *Tsc1*^-/-^ primary hepatocytes were transfected with miR-329-3p ASO for 48 h, respectively. Scale bar = 100 μm. Right, quantitative determination of Pi^+^ KCs among groups as indicated, *n* = 3. **m** Western blot assays showing the SGMS1 abundance in KCs transfected with the mimic or ASO for miR-329-3p. **n** SGMS1-3′ UTR luciferase activity assays using HEK293 cells transfected with control mimic or miR-329-3p mimic and control ASO or miR-329-3p ASO for 24 h. *p < 0.05.

Accumulating evidence indicates that microRNAs (miRNAs) play a crucial role in mediating cell-cell interaction. To determine whether certain miRNAs mediate the KC death promoted by mTORC1-activated hepatocytes, we first analyzed the miRNA expression profile of mouse liver exposed to Con-A for 2 h (GSE 76345). After Con-A injection, 23 miRNAs were significantly upregulated in the mouse liver (Fig. 3a). We selected candidate target genes related to “Apoptotic process” by using TargetScan and DAVID databases (Fig. 3b), and we selected the gene *Sgms1* because SGMS1 is a critical enzyme in the final step in the production of sphingomyelin (SM) [21], which is synthesized by the transfer of phosphocholine from phosphatidylcholine to ceramide, a well-established apoptosis inducer (Fig. 3c) [22]. SGMS1 abundance was reduced in KCs treated with *Tsc1*^-/-^ hepatocyte-derived CM (Fig. 3d, e), and knocking down *Sgms1* enhanced KC death (Fig. 3f, g). To verify the role of ceramide production in KC death, KCs deficient for protein phosphatase 2 A catalytic subunit α (PP2Acα) were incubated with *Tsc1*^-/-^ hepatocyte-derived CM for 48 h: PP2A cα ablation in KCs attenuated the cell death induced by *Tsc1*^-/-^ hepatocyte-derived CM (Supplementary Fig. 4). Together, our results demonstrate a pro-survival role for SGMS1 in KCs.

Three miRNAs can regulate *Sgms1* expression (Fig. 3h), and the abundance of one of these miRNAs, miR-329-3p, was significantly increased in both *Tsc1* deficient hepatocytes (Fig. 3i) and livers exposed to Con-A (Fig. 3j). but those of the other two miRNAs (miR-124-3p and miR-804) remained unchanged in *Tsc1*^-/-^ hepatocytes relative to the levels in *Tsc1*^+/+^ hepatocytes (Supplementary Fig. 5). Notably, transfection of miR-329-3p mimics enhanced cell death (Fig. 3k) and transfection of an antisense oligonucleotide (ASO) of the miRNA diminished cell death in KCs incubated with *Tsc1*^-/-^ hepatocyte-derived CM (Fig. 3l). Together, these data indicate that the hepatocytes in which mTORC1 is activated might upregulate miR-329-3p expression and promote KC loss in acute hepatitis.

In KCs, transfection of miR-329-3p mimics reduced SGMS1 abundance but transfection of the ASO increased SGMS1 abundance (Fig. 3m). We found that the SGMS1 3′-untranslated region (UTR) and the miR-329-3p seed sequence were almost perfectly paired (Supplementary Fig. 6). The results of luciferase reporter assays confirmed the activation of miR-329-3p in the pSI-check2-SGMS1-3′-UTR luciferase construct (Fig. 3n). These results collectively indicate that mTORC1-activated hepatocytes might produce and secrete exosome-enveloped miR-329-3p and thereby downregulate SGMS1 expression in KCs and promote KC death.

### Activation of mTORC1 in KCs promotes Con-A-induced hepatitis

**Fig. 4:**
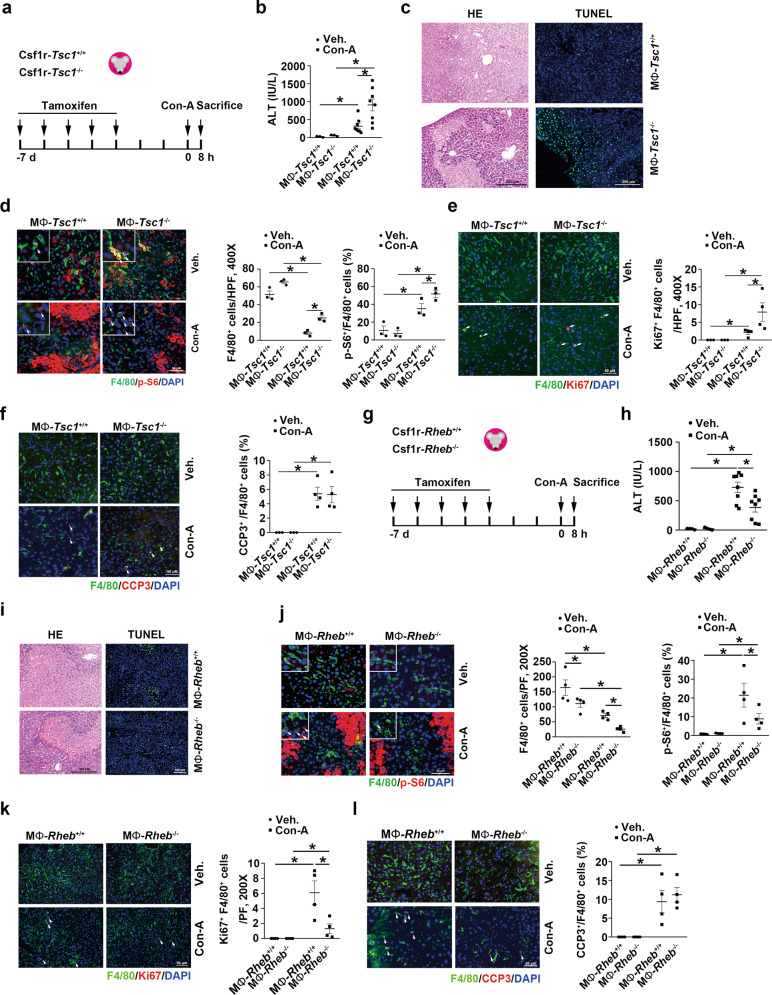
Activation of mTORC1 in KCs aggravates Con-A induced hepatitis. **a** The strategy for inducing macrophage *Tsc1* deletion in Csf1r-Cre^+/−^, *Tsc1*^fl/fl^ mice and Con-A administration. **b** The ALT levels in serum of mice exposed to Con-A for 8 h. Veh. *n* = 3, Con-a *n* = 7. **c** Representative HE and TUNEL- stained mouse livers. Scale bar = 200 μm as indicated in figures. **d** Left, representative immunofluorescent staining images for F4/80 with p-S6 (white arrows). Scale bar = 50 μm. Middle, quantitative determination of F4/80^+^ cells among groups as indicated, *n* = 3. Right, quantitative determination of F4/80^+^ and p-S6^+^ cells among groups as indicated, *n* = 3. **e** Left, representative immunofluorescent staining images for F4/80 with Ki67 (white arrows). Scale bar = 50 μm. Right, quantitative determination of F4/80^+^ and Ki67^+^ cells among groups as indicated. Veh. *n* = 3, Con-A *n* = 4. **f** Left, representative immunofluorescent staining images for F4/80 with CCP3 (white arrows). Scale bar = 50 μm. Right, quantitative determination of F4/80^+^ and CCP3^+^ cells among groups as indicated. Veh. *n* = 3, Con-A *n* = 4. **g** The strategy for inducing macrophage *Rheb* deletion in Csf1r-Cre^+/−^, *Rheb*^fl/fl^ mice and Con-A administration. **h** The ALT levels in serum. Veh. *n* = 3, Con-A *n* = 7. **i** Representative HE and TUNEL- stained mouse livers. Scale bar = 200 μm or 100 μm as indicated in figures. **j** Left, representative immunofluorescent staining images for F4/80 with p-S6 (white arrows) and quantification. Scale bar = 50 μm. *n* = 4. **k** Left, representative immunofluorescent staining images for F4/80 with Ki67 (white arrows). Scale bar = 50 μm. Right, quantitative determination of F4/80^+^ and Ki67^+^ cells among groups as indicated, *n* = 4. **l** Left, representative immunofluorescent staining images for F4/80 with CCP3 (white arrows). Scale bar = 50 μm. Right, quantitative determination of F4/80^+^ and CCP3^+^ cells among groups as indicated, *n* = 4. ^*^p < 0.05.

To investigate the role of mTORC1-activated KCs on the injury of hepatocytes during immune mediated hepatitis, we generated two mouse models with macrophage-specific *Tsc1* deficiency (MΦ-*Tsc1*^-/-^ mice, Fig. 4a and Supplementary Fig. 7) and macrophage-specific ablation of *Rheb1* (MΦ-*Rheb*^-/-^ mice, Fig. 4g). At 8 h after Con-A injection, the MΦ-*Tsc1*^-/-^ mice exhibited, relative to control littermates, higher plasma ALT levels, more severe liver injury, and increased hepatocyte death and KC accumulation (Fig. 4b–d). As compared with control littermates, the MΦ-*Tsc1*^-/-^ mice showed enhanced KC proliferation, comparable KC death (Fig. 4e, f), and increased neutrophil infiltration, similar CD3^+^ T-cell and CD11b^+^ monocyte infiltration after Con-A injection (Supplementary Fig. 8a–c). At 8 h after Con-A injection, the MΦ-*Rheb*^-/-^ mice showed, relative to control littermates, lower plasma ALT levels, diminished liver injury, and decreased hepatocyte death (Fig. 4h, i). Furthermore, in the MΦ-*Rheb*^-/-^ liver after Con-A injection, we observed diminished KC accumulation, decreased KC proliferation, and similar KC death as compared with that in control littermates (Fig. 4j–l), and we also detected decreased neutrophil, T-cell, and CD11b^+^ monocyte infiltration in the MΦ-*Rheb*^-/-^ mice (Supplementary Fig. 8d–f). These data suggest that the activation of mTORC1 in KCs aggravates Con-A induced hepatitis.

To further investigate the role and mechanisms of action of KCs in hepatocyte injury, primary cultured KCs isolated from wild-type (WT), *Tsc1*^fl/fl^, and *Rheb*^fl/fl^ mice were treated with tamoxifen to induce the ablation of the respective target genes; subsequently, primary cultured WT hepatocytes were incubated with KC-derived CM for 48 h (Supplementary Fig. 9a). Our results showed that hepatocyte death was diminished by *Tsc1*^-/-^ KC-derived CM but enhanced by *Rheb*^-/-^ KC-derived CM (Supplementary Fig. 9b–e). Taken together, specific activation of mTORC1 promotes the KCs proliferation. The increased KCs aggravate Con-A-induced hepatitis by promoting inflammatory cells infiltration and activation, but not by affecting hepatocyte survival directly.

### mTORC1-activated KCs produce CFB and thus exacerbate hepatocyte death and Con-A induced hepatitis

**Fig. 5:**
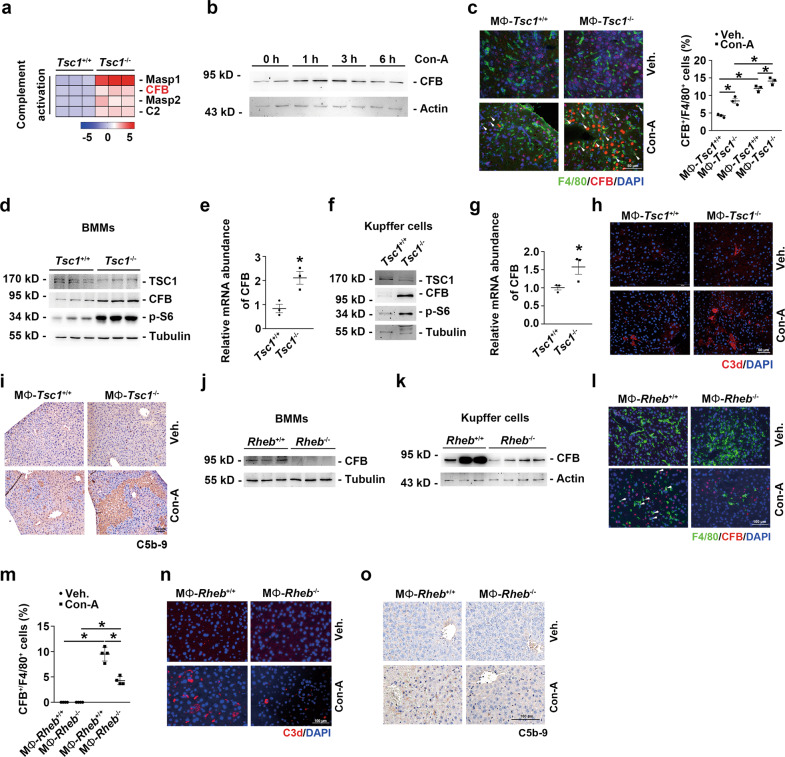
The activation of mTORC1 in KCs enhances complement alternative system. **a** Expression heatmap of genes of neutrophils chemotaxis or complement activation analyzed by RNA-seq from *Tsc1*^+/+^ and *Tsc1*^-/-^ BMMs (*n* = 3 each). **b** Western blotting result was shown the expression of CFB protein in hepatic tissues from mice. **c** Left, representative co-immunofluorescent staining images for F4/80 with CFB. Scale bar = 50 μm. Right, quantitative determination of F4/80^+^ and CFB^+^ cells among groups as indicated, *n* = 3. **d** Western blotting assay showing the abundance for TSC1, CFB, and p-S6 in BMMs. **e** qRT-PCR analysis showing the CFB mRNA abundance in BMMs, *n* = 3. **f** Western blotting assay showing the abundance for TSC1, CFB, and p-S6 in KCs. **g** qRT-PCR analysis showing the CFB mRNA abundance in KCs, *n* = 3. **h** Representative immunofluorescent staining images for C3d. Scale bar = 50 μm. **i** Representative immunostaining images for C5b-9. Scale bar = 50 μm. **j** Western blotting assay showing the abundance for Rheb and CFB in BMMs. **k** Western blotting assay showing the abundance for Rheb and CFB in KCs. **l** Representative co-immunofluorescent staining images for F4/80 with CFB (white arrows). Scale bar = 100 μm. **m** Quantitative determination of F4/80^+^ and CFB^+^ cells among groups as indicated, *n* = 3. **n** Representative immunofluorescent staining images for C3d. Scale bar = 100 μm. **o** Representative immunostaining images for C5b-9 among groups as indicated. Scale bar = 100 μm. ^*^p < 0.05.

Activated KCs secrete a large variety of complement factors after liver injury [23]. To clarify the mechanisms of action of mTORC1-activated KCs in hepatitis, we analyzed the differentially expressed genes related to the complement system in *Tsc1*^-/-^ KCs (Fig. 5a). CFB, one of the essential molecules for activating the complement alternative pathway [24], was upregulated at 1 and 3 h and then declined to the basal level at 6 h in the mouse liver after Con-A injection (Fig. 5b). In *Tsc1*^-/-^ macrophages, CFB abundance was clearly increased as compared with the level in control cells (Fig. 5a, c). *Tsc1* ablation in both primary cultured bone marrow-derived macrophages (BMMs) and KCs upregulated CFB expression (Fig. 5d–g). Moreover, C3d and C5b-9 deposition in the liver was considerably higher in MΦ-*Tsc1*^-/-^ mice than in MΦ-*Tsc1*^+/+^ mice after Con-A injection (Fig. 5h, i). Conversely, ablation of *Rheb* in BMMs or KCs downregulated CFB expression (Fig. 5j, k), and the number of CFB^+^ KCs and the deposition of C3d and C5b-9 in the liver of MΦ-*Rheb*^-/-^ mice were less than that in control littermates (Fig. 5l–o).

**Fig. 6:**
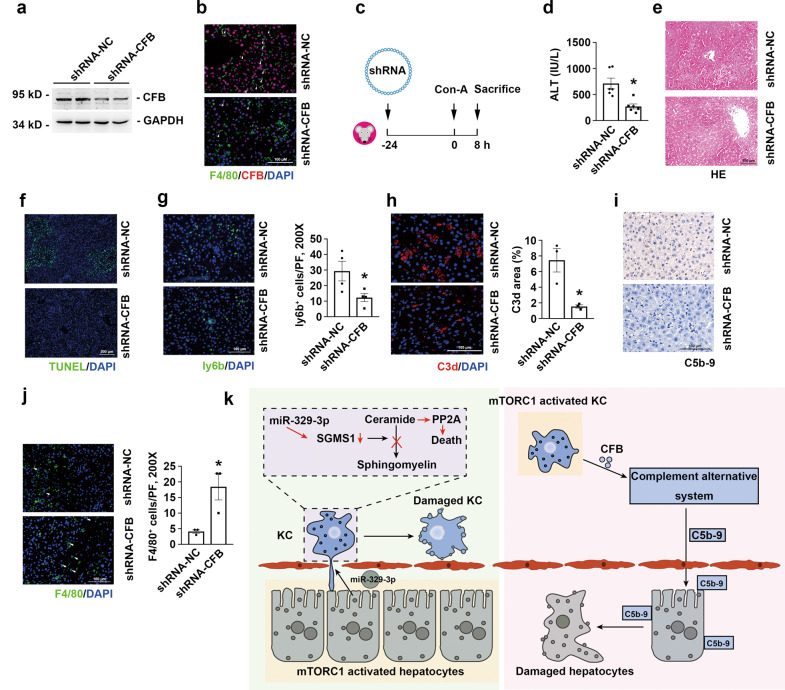
Down regulation of CFB in liver protects against Con-A induced liver injury. **a** Western blotting assay showing CFB expression in mouse livers after shRNA-CFB injection. **b** Representative co-immunofluorescent staining images for F4/80 with CFB (white arrows). Scale bar = 100 μm. **c** The strategy for establishing a mouse model of injection of shRNA-CFB and Con-A administration. **d** The ALT levels in serum of mice exposed to Con-A for 8 h, *n* = 6. **e** Representative HE-stained mouse livers. Scale bar = 100 μm. **f** Liver sections of were immunofluorescent stained with TUNEL. Scale bar = 200 μm. **g** Left, representative immunofluorescent staining images for ly6b. Scale bar = 100 μm. Right, quantitative determination of ly6b^+^ cells among groups as indicated, *n* = 4. **h** Left, representative immunofluorescent staining images for C3d. Scale bar = 100 μm. Right, quantitative determination of C3d area in a field of vision, *n* = 4. **i** Representative immunostaining images for C5b-9. Scale bar = 100 μm. **j** Left, representative immunofluorescent staining images for F4/80 (white arrows). Scale bar = 100 μm. Right, quantitative determination of F4/80^+^ cells among groups as indicated, *n* = 4. *p < 0.05. **k** Schematic working model on the role of mTORC1 activation in hepatocytes and KCs in the pathogenesis of ALD.

To evaluate whether CFB induction drives Con-A-induced liver injury, mice were injected with shRNA-CFB through the tail vein to knockdown CFB expression and then injected with Con-A (Fig. 6a–c). Knocking down CFB largely decreased plasma ALT levels, attenuated liver injury, and diminished cell death in mice injected with Con-A (Fig. 6d–f), and the knockdown also markedly alleviated neutrophil accumulation and activation of the complement alternative system in the liver (Fig. 6g–i). The number of KCs was increased, which might be related to the rapid replenishment of monocyte-derived KCs (Fig. 6j). Taken together, these data indicate that mTORC1 activation in KCs might upregulate CFB expression and thereby promote hepatocyte death and liver injury.

## Discussion

Although the loss of KCs and hepatocytes is known to represent a common pathogenic phenomenon in hepatitis, the mechanisms of cell-death regulation in hepatitis remain poorly understood. Here, we identified mTORC1 as a “double-edged sword” in liver injury in the very early stage of the disease in the murine hepatitis model: Whereas mTORC1-activated hepatocytes secrete miR-329-3p to promote KC death, which results in an alleviation of the inflammatory response and liver injury, mTORC1-activated KCs express increased levels of CFB proteins to activate the complement alterative pathway, which aggravates hepatocyte death and liver injury (Fig. 6k).

Unstimulated hepatocytes are reported to release a factor—which is >10 kDa in size and is inactivated by boiling—that drastically enhances the ability of KCs to respond to endotoxin challenge [25, 26]. Unstimulated KCs co-cultured with hepatocytes act as a feeder layer and promote or maintain hepatocyte protein synthesis [27, 28]. Here, we found that the CM from WT hepatocytes promoted KC survival as compared to the complete medium, whereas the CM from WT KCs maintained the survival of hepatocytes. These findings consistently showed that the microenvironmental crosstalk between hepatocytes and KCs is essential for the maintenance of liver homeostasis.

The role of mTORC1 in liver is controversial. Rapamycin was reported to prevent Con-A-induced hepatitis by inhibiting lymphocyte activation [29]. However, the activation of mTORC1 in hepatocytes protects liver from I/R-induced injury dependent on NF-κB proinflammatory cytokine signaling pathway [30]. We observed here that mTORC1 signaling in hepatocytes was activated as early as 1 h after Con-A challenge and then declined from 3 to 8 h. while, mTORC1 in KCs was activated at 1 h and the activation lasted until 8 h, and the mTORC1-activated KCs could lead to severe liver injury. In accordance with previous studies, we speculate that pre-treatment with rapamycin inhibits mTORC1 activation in KCs rather than in hepatocytes, and the loss of KCs might represent a protective event in the early stage of acute hepatitis to alleviate the inflammatory response.

Besides mTORC1, mTORC2 is another complex which mediated mTOR signals. Previous study showed that rapamycin partially alleviates I/R-induced liver injury through the promotion of mTORC2 activity [31], and the deficiency of Rictor aggravates hepatic I/R injury by suppressing autophagy [32]. It was reported that mTORC1 inhibits mTORC2 signaling through phosphorylating Sin1 [33]. Therefore, it is hard to exclude the protected role of mTORC2 in hepatocytes on the Con-A-induced hepatitis.

Among the abundant miRNAs in hepatocytes, miR-329-3p was confirmed to be involved in cell proliferation [34, 35]. To our knowledge, miR-329-3p regulation of SGMS1 is a previously undescribed event occurring in the progression of liver disease. SGMS functions in cell death and survival by regulating ceramide and diacylglycerol levels [36]. Serine and fatty acyl CoA are condensed to generate ceramide in the endoplasmic reticulum (ER) [37], and ceramide is then further metabolized to yield SM by SGMS1 in the Golgi apparatus [21, 38]. Ceramide accumulation contributs to cell death in macrophages [39]; specifically, C16 ceramide elicits triacylglycerol-induced apoptosis in macrophages by activating the mitochondrial apoptosis pathway [40]. In animal models of non-alcoholic fatty liver disease, the expression of acid sphingomyelinase is increased [41], and whereas total hepatic ceramides are not altered, a change occurs in the relative abundance of ceramides, from higher levels of C24 ceramides to higher levels of C16 ceramides [41]. Ceramide was described as a PP2A activator, and accordingly, the increased cell death induced by *Tsc1*^-/-^ CM was diminished in *Ppp2ca*^-/-^ KCs. These findings indicate that the miR-329-3p-induced KC death depends on C16-ceramide accumulation. However, we did not detect any changes in C16-ceramide concentration in KCs after Con-A treatment due to limited conditions, and thus the distinct roles of C24 and C16 ceramides in PP2A activation remain unknown.

SM is a type of sphingolipid that plays a crucial role in the composition of plasma lipoproteins and biological membranes [42]. SGMS1, the major SGMS in macrophages [43], plays a critical role in the cell growth of mouse lymphoid cells [21], and *Sgms*^-/-^ mice show moderate neonatal lethality and severe pancreatic dysfunction [44]. SGMS1 deficiency also exerts no effect on total ceramide levels, but substantially decreases SM levels in the plasma membrane of macrophages and other cells [43]. CD4^+^ T cells from *Sgms1*^-/-^ mice show severe deficiency of membrane SM and proliferation [45]. Thus, the targeting of SGMS1 by miR-329-3p potentially represents a leading mechanism by which the SM level is affected in KCs. This agrees with our results showing that the deficiency of SGMS1 induced by miR-329-3p rendered KCs susceptible to death. Moreover, *Sgms1*^-/-^ mice were reported to show diminished sensitivity to Con-A-induced hepatitis [45]. Therefore, the concentration of SM or C16 ceramide in KCs could be used to predict KC survival, and the targeting of SGMS1 in KCs could reduce hepatocyte damage.

The complement system is one of the first lines of defense against microbial infection, which eventually leads to the formation of the cytolytic membrane attack complex (C5b-9) [46]. Furthermore, soluble complement fragments (such as C3a and C5a) are potent chemoattractants for various leukocytes, such as neutrophils [47]. Accordingly, in acetaminophen-induced hepatitis, we observed neutrophil accumulation in areas that were negative for hepatocytes enriched in p-S6 (data not shown). Furthermore, C5b-9 deposition is observed in patients with either non-alcoholic steatohepatitis or alcoholic hepatitis [48, 49]. mTORC1 activation in KCs enhanced the expression of CFB and activated the complement alternative pathway in the liver, which was confirmed by the increased immunostaining of C3d and C5b-9 deposited in the mouse liver with macrophage-specific *Tsc1* deficiency. Thus, in terms of their functions, KCs not only support inflammation and control infection, but also damage hepatocytes by activating the complement system.

In conclusion, our findings collectively demonstrate that mTORC1 activation is a key event in the early stage of hepatitis. Our study has not only explained the mechanism of KC loss at the molecular level, but also revealed the role of the remaining KCs. The remaining KCs could potentially determine the disease outcome in patients, and this could form the basis for the development of new interventions against hepatitis.

## Materials and methods

### Animals

All animals were maintained in the specific pathogen-free Laboratory Animal Center of Nanjing Medical University according to the guidelines of the Institutional Animal Care and Use Committee at Nanjing Medical University, Nanjing, China.

Male C57BL/6 mice weighing 18–22 g were acquired from the specific pathogen-free Laboratory Animal Center of Nanjing Medical University.

Homozygous *Tsc1* floxed mice (005680, The Jackson Laboratory, Bar Harbor, ME) were ordered from Jackson Laboratory. *Rheb* floxed mice were kindly provided by Dr. Bo Xiao (Southern University of Science and Technology, China) [18, 19]. Mice expressing the tamoxifen-inducible MerCreMer fusion protein under the control of macrophage specific mouse Csf1r promoter were ordered from Jackson Laboratory (019098, FVB-Tg(Csf1r-cre/Esr1*)1Jwp/J, donated from Jeffrey Pollard, The Jackson Laboratory, Bar Harbor, ME) [50]. Alb-Cre mice (003574, B6.Cg-Speer6-ps1Tg(Alb-cre)21Mgn/J, The Jackson Laboratory, Bar Harbor, ME) were ordered from Jackson Laboratory. Male and female mice between 6 and 8 weeks of age were used in this study.

### Animal models

For Con-A models, a single dose of Con-A (Sigma-Aldrich, St. Louis, MO) was injected at 15 mg/kg through the tail vein to induce acute hepatitis in mice. Animals were sacrificed to perform analysis from 1–8 h after the injection randomly with random number table. Blood and liver samples were collected.

To knock down the expression of CFB, shRNA-CFB (ordered from Ruizhen, Nanjing) was performed. A solution of shRNA-CFB or vector plasmids was prepared in PBS. Animals were divided into two groups randomly. Mice were injected with shRNA-CFB or vector plasmids by tail vein at 1 mg/kg to induce CFB downregulation. Twenty-four hours after shRNA-CFB injection Con-A was administered intravenously at 15 mg/kg to all animals. After 8 h animals were sacrificed. Blood and liver samples were collected.

Plasma concentrations of alanine transaminase (ALT) were measured using commercially available kits from Nanjing Jiancheng Bioengineering Institute (C009-2).

### Histology and immunohistochemistry

For paraffin staining, livers were fixed in 10% formaldehyde and paraffin-embedded. Three μm sections were cut and deparaffinized, rehydrated, and boiled at 100 °C. Liver sections were stained with hematoxylin-and-eosin (HE), periodic acid-Schiff (PAS) or immunolabeled with C5b-9 (cat: ab55811, Abcam). The staining of human samples was using TSA-RM-275 kit (Panovue, China).

For cryosections, livers were frozen in Optimum Cutting Temperature (O.C.T.) compound. Three μm sections were cut and immunolabelled with primary antibodies specifically binding F4/80 (cat: 14–4801-82, Invitrogen, USA), p-S6 (Ser235/236) (cat: 4858, Cell Signaling Technology, USA), CD11b (cat: 557396, BD Biosciences), CD3 (cat: 555273, BD Biosciences), ly6b (cat: MCA771G, Bio-Rad, California, USA), Ki67(cat: ab15580, Abcam, Cambridge, UK), cleaved Caspase 3 (cat: 9664, Cell Signaling Technology, USA), CFB (cat: NBP1-89985, Novus; cat: ab192577, Abcam, Cambridge, UK), C3d (cat: AF2655, R&D Systems) or TdT-mediated dUTP-biotin nick end labeling (TUNEL, Promega, Madison, WI).

Images were acquired with a OLYMPUS DP74 and BX53 Epifluorescence microscope. Original pictures were adjusted for brightness, contrast, and color balance and post processed using Adobe Photoshop CS6. Cell counting was performed using Adobe Photoshop CS6.

### Isolation of hepatocytes, KCs and BMMs

Hepatocytes or KCs were isolated following a previously established method [51]. Briefly, the livers were perfused in situ with 15–20 ml Hank’s balanced salt solution (HBSS) buffer containing 0.5 mM EGTA for 10 min, and 15–30 ml HBSS containing 1 mM calcium and 32 mg collagenase (cat: 9001-12-1, Gibco) until the liver was sufficiently digested. Released the hepatic cells gently by cutting the liver lobes and continue until all of the big clumps were gone. Centrifuged for 3 min at a speed of 50 × *g* at 4 °C. Collected the supernatant containing the KCs. The hepatocytes were in the pellet. For hepatocytes, gently resuspended the hepatocyte pellet in 30 mL of HBSS- CaCl_2_. Centrifuged for 3 min at 50 × *g* at 4 °C. Aspirated the rinse buffer supernatant, hepatocytes were seed in DMEM containing 10% FBS. Six hours later, the cells from floxed mice were treated with adenovirus carrying Cre recombinase for 48 h to get gene deletion. For KCs, the supernatant containing KCs was separated by centrifugation in Percoll gradient. The KC-enriched fraction located at the interface of the 25–50% Percoll layer was seeded in RPMI 1640 containing 10% FBS. KCs from floxed mice were treated with 1 μM 4-Hydroxytamoxifen (H6278, Sigma-Aldrich) for 7 days from the beginning of the culture to get gene deletion.

Bone marrow cells were flushed out of the tibias and femur bones with a syringe filled with DMEM media. The bone marrow cells were cultured with DMEM containing 10% FBS and 10 ng/ml of mouse M-CSF (cat: 416-ML-050, R&D) for 7 days to obtain bone marrow derived-macrophages (BMMs). BMMs from floxed mice were treated with 1 μM 4-Hydroxytamoxifen for 7 days from the beginning of the culture to get gene deletion.

### Cell transfection

For miRNA mimic or inhibitor, synthetic miRNA duplexes were synthesized (IBSBIO, China) and transfected, as previously described [52]. Briefly, HEK293 cells in each well (6-well plates) were transiently transfected with 200 pmoles of miR-329-3p mimic or control mimic, or 200 pmoles of miR-329-3p antisense oligonucleotide (ASO) or respective control NC using Lipofectamine 3000 reagent (Invitrogen, USA). For siRNA, Raptor siRNA oligos, SGMS1 siRNA oligos and NC were synthesized (IBSBIO, China) and transfected into hepatocytes or KCs using Lipofectamine 3000 reagent according to the manufacturer’s instruction.

### 3’UTR luciferase assays

The miRNA 3′UTR target clone (Luc-SGMS1-3’UTR, binding site sequence; GTGTGT) was purchased from HANBIO (Shanghai, China), which contains firefly luciferase as internal control fused downstream to renilla luciferase. The HEK293 cells were co-transfected with Luc-SGMS1-3′UTR luciferase or control vector, and miR-329-3p mimic (or inhibitor) or its respective control using Lipofectamine 3000 reagent. At 24 h after transfection, renilla and firefly luciferase activities were measured using Dual Luciferase Reporter Assay Kit (cat: DL101-01, Vazyme, Nanjing, China) according to the manufacturer’s protocols. The target sites within SGMS1 3’UTR reporter were mutated to generate a mutant reporter construct (5′-CACACA-3′, bolds indicate mutations), which was used as a negative control.

### Conditioned media (CM) from cultural primary cells

Primary hepatocytes or KCs from floxed mice were treated for 48 h as indicated. Cultural media was harvested and centrifuged (1500 rpm for 5 min at 4 °C). The supernatant was aliquot and stored at −80 °C for the subsequent experiments.

### Exosomes isolation

Exosomes were prepared from CM of primary hepatocytes (1 × 10^7^ cells/10 cm dish) as previously described [53]. CM was first spun at 320 x g for 10 min followed by centrifugation at 2000 x *g* for 15 min at 4 °C to remove debris and dead cells, and then underwent a 10,000 x g centrifugation step for 30 min at 4 °C. The supernatant was centrifuged at 100 000 *g* for 70 min at 4 °C. Finally, the resulting pellet, which contained exosomes, was washed once in PBS and collected by ultracentrifugation at 100,000 x *g* for 70 min at 4 °C.

### Western blot analysis

Primary cultured KCs and BMMs were lysed in 2× sample buffer. Liver tissues were lysed with RIPA solution containing 1% NP40, 0.1% SDS, 100 μg/ml PMSF, 1% protease inhibitor cocktail, and 1% phosphatase I and II inhibitor cocktails (Sigma, St Louis, MO) on ice for 30 min. The supernatants were collected after centrifugation at 13,000 *g* at 4 °C for 15 min. Protein concentration was determined by the bicinchoninic acid protein assay (BCA Kit; Pierce Thermo-Scientific, Rock- ford, IL) according to the manufacturer’s instruction. Equal amount of protein for each sample was loaded into sodium dodecyl sulfate-polyacrylamide gel electrophoresis and transferred onto (nitrocellulose filter membrane) NC membranes. The membranes were probed with antibodies against: anti-p-S6 (cat: 4858, Cell Signaling Technology), anti-S6 (cat: 2217, Cell Signaling Technology), anti-Rheb1 (cat: ab25873, Abcam), anti-p-p70 S6K (Thr421/Ser424) (cat: 9204 L, Cell Signaling Technology), anti-p70 S6K (cat: 9202, Cell Signaling Technology), anti-p-4EBP1 (Thr37/46) (cat: 2855, Cell Signaling Technology), anti-4EBP1 (cat: 9452, Cell Signaling Technology), anti-Raptor (cat: 2280 S, Cell Signaling Technology), anti-TSC1 (cat: 4906 S, Cell Signaling Technology), anti-CFB (cat: ab192577, Abcam), anti-SGMS1 (cat: sc-166436, Santa Cruz Biotechnology), anti-β-actin (cat: sc-47778, Santa Cruz Biotechnology), anti-GAPDH (cat: sc-25778, Santa Cruz Biotechnology) and anti-α-tubulin (cat: T9026, Sigma, St. Louis, MO), respectively.

### Reverse transcription and quantitative PCR assays

Total RNA was extracted with TRIzol (Invitrogen, Carlsbad, CA, USA). For assays of mRNA, 1 μg of cDNA was generated using the HiScript^®^ II Q RT SuperMix kit (cat: R223-01, Vazyme, Nanjing, China). Real-time qRT-PCR assay was carried out using Roche Light Cycler 96 Systems and 96-well optical reaction plates (Roche, Mannheim, Germany). For qRT-PCR assays for miRNA, the miRNA 1st Strand cDNA Synthesis Kit (by stem-loop) (cat: MR101-02, Vazyme, Nanjing, China) was used to reverse transcribe the total RNA, according to the manufacturer’s instructions. In brief, 1 μg of RNA was added to 2 μl of 5× gDNA Wiper Mix, and RNase-free water was added to a total volume of 10 μl. The mixture was then incubated at 42 °C for 2 min. Stem-loop primer (1 μl), 2 μl of 10× RT Mix, 2 μl of HiScript II Enzyme Mix, and 5 μl of RNase-free water were added to the reaction mix. The total reaction mixture was incubated at 25 °C for 5 min, then at 50 °C for 15 min, and at 85 °C for 5 min. The stem-loop primer and qRT–PCR primers were synthesized by IBSBIO (Shanghai, China). Real-time qRT-PCR assay was carried out using Roche Light Cycler 96 Systems and 96-well optical reaction plates (Roche, Mannheim, Germany). Mice U6 snRNA was the reference RNA used for normalization. The results were analyzed with the 2 − ΔΔCt method.

### Microarray profiling and bioinformatic predictions

Heatmap data were extracted from the miRNA array database (GSE 76345) available in public domain [54]. To identify differentially expressed miRNAs with statistical significance, we performed a volcano plot filtering. The threshold to screen dysregulated miRNAs is log_2_fc ≥2 and *p* < 0.05. The target gene of miRNAs was predicted based on the overlap of prediction results by four out of three target prediction tools, including TargetScan, miRbase, miRTarBase, TransmiR.

### Expression profiling by array

Total RNA samples were extracted from BMMs of MΦ-*Tsc1*^+/+^ or MΦ-*Tsc1*^-/-^ mice. Analysis was performed by CapitalBio Technology (Beijing, China) and available in pubic domain (https://www.ncbi.nlm.nih.gov/geo/query/acc.cgi?acc=GSE218700).

### Flow cytometry

Hepatic MNCs were isolated from the mice at 3 h and 8 h after Con-A treatment, and incubated with FCR blocker (cat: 130-092-575, Miltenyi Biotec) for 20 min in order to prevent nonspecific binding. The cells were stained with various antibodies, including fluorescein isothiocyanate (FITC)- conjugated anti-mouse CD11b (clone M1/70, cat: 101206, BioLegend), APC-conjugated anti-mouse F4/80 (clone BM8, cat: 123115, BioLegend). Cells were detected and analyzed with Beckman Coulter Navios Flow Cytometer and the Kaluza.

### Statistics

All data examined are presented as mean ± SEM. Statistical analyses were performed using the Graphpad Prism 8 (GraphPad Software, San Diego, CA). Comparison between groups was made using one-way analysis of variance, followed by the Student *t*-test. *p* < 0.05 was considered as statistically significant.

## Supplementary information

Original western blots

supplementary figures

checklist

## Data Availability

All data generated or analyzed during this study are included in this published article.
