# Supplementary figures and images for: Mechanistic target of rapamycin complex 1 orchestrates the interplay between hepatocytes and Kupffer cells to determine the outcome of immune-mediated hepatitis
Source: Cell Death Dis. 2022 Dec 9;13(12):1031. doi: 10.1038/s41419-022-05487-0 (PMC9734196; doi:10.1038/s41419-022-05487-0)

Original western blots

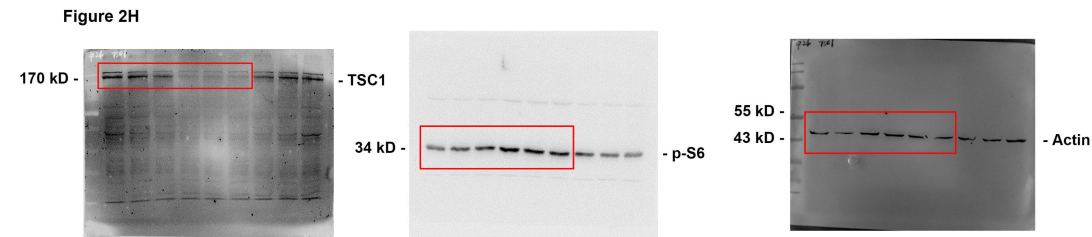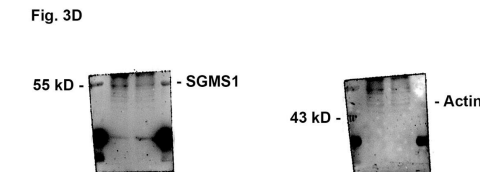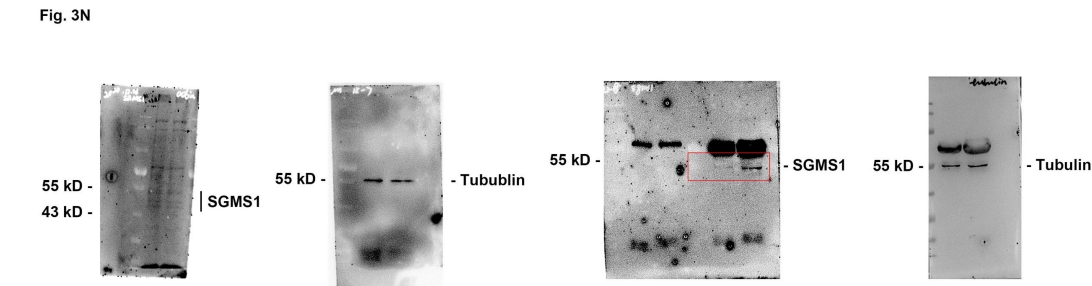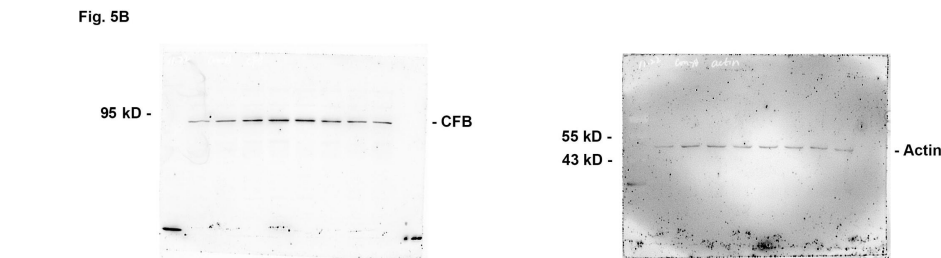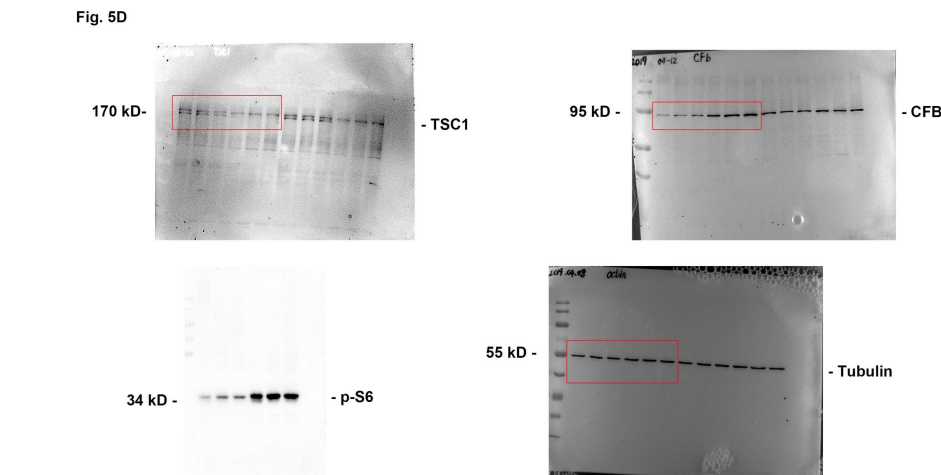

Fig. 5F

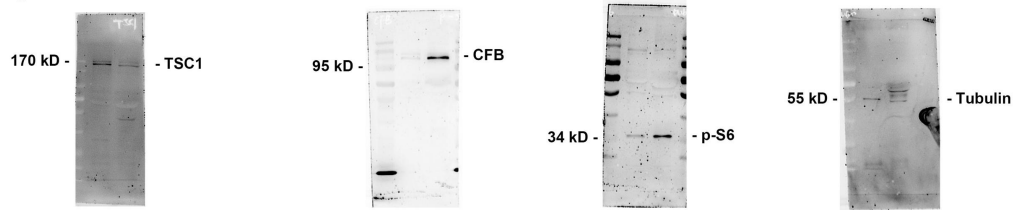

Fig. 5J

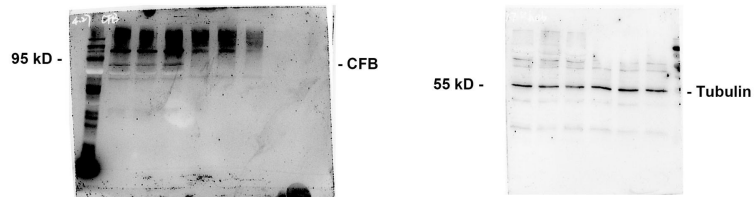

Fig. 5K

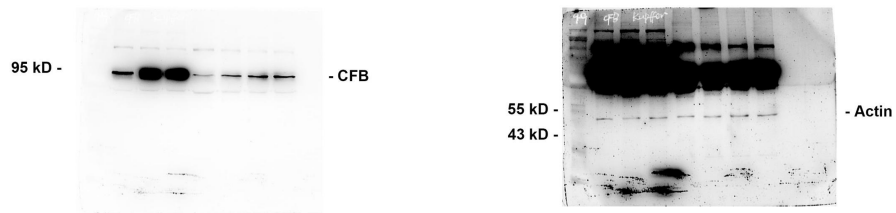

Supplementary Fig. 1c

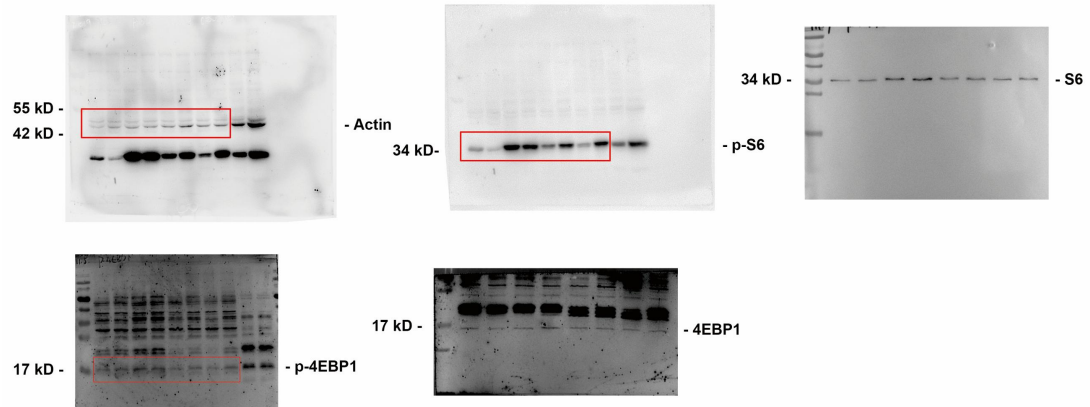

Supplementary Fig.3b

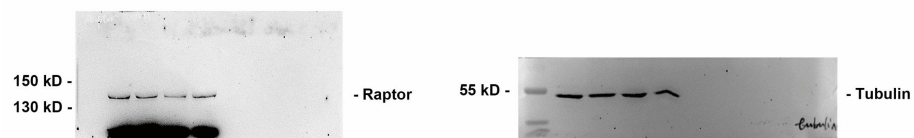

Supplement: Supplementary file 1 — Original western blots [file 41419_2022_5487_MOESM1_ESM.pdf]
