# Supplementary material for: Mechanistic target of rapamycin complex 1 orchestrates the interplay between hepatocytes and Kupffer cells to determine the outcome of immune-mediated hepatitis
Source: Cell Death Dis. 2022 Dec 9;13(12):1031. doi: 10.1038/s41419-022-05487-0 (PMC9734196; doi:10.1038/s41419-022-05487-0)
Supplement: Supplementary file 2 — supplementary figures [file 41419_2022_5487_MOESM2_ESM.pdf]

Supplementary Materials and Methods

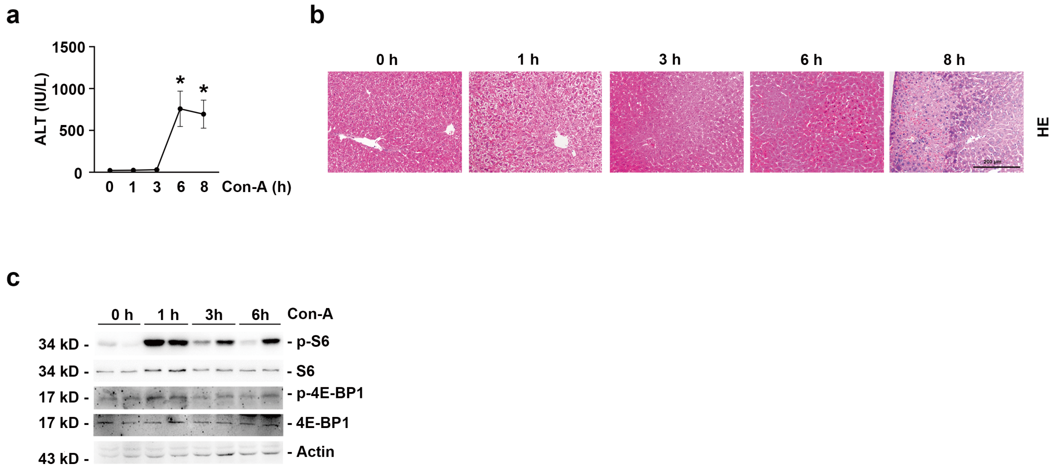

Supplementary Fig. 1 Time course analysis of Con-A-induced liver injury. WT mice were treated with Con-A (15 mg/kg) for 1 h, 3 h, 6 h and 8 h. **a** The ALT levels in serum of mice, n = 4. **b** Representative HE-stained mouse livers. Scale bar = 200 μm. **c** Representative western blotting result of mTORC1 signaling in hepatic tissues. * p<0.05. Data are expressed as the mean ± SEM, significance was determined using the 1-way ANOVA.

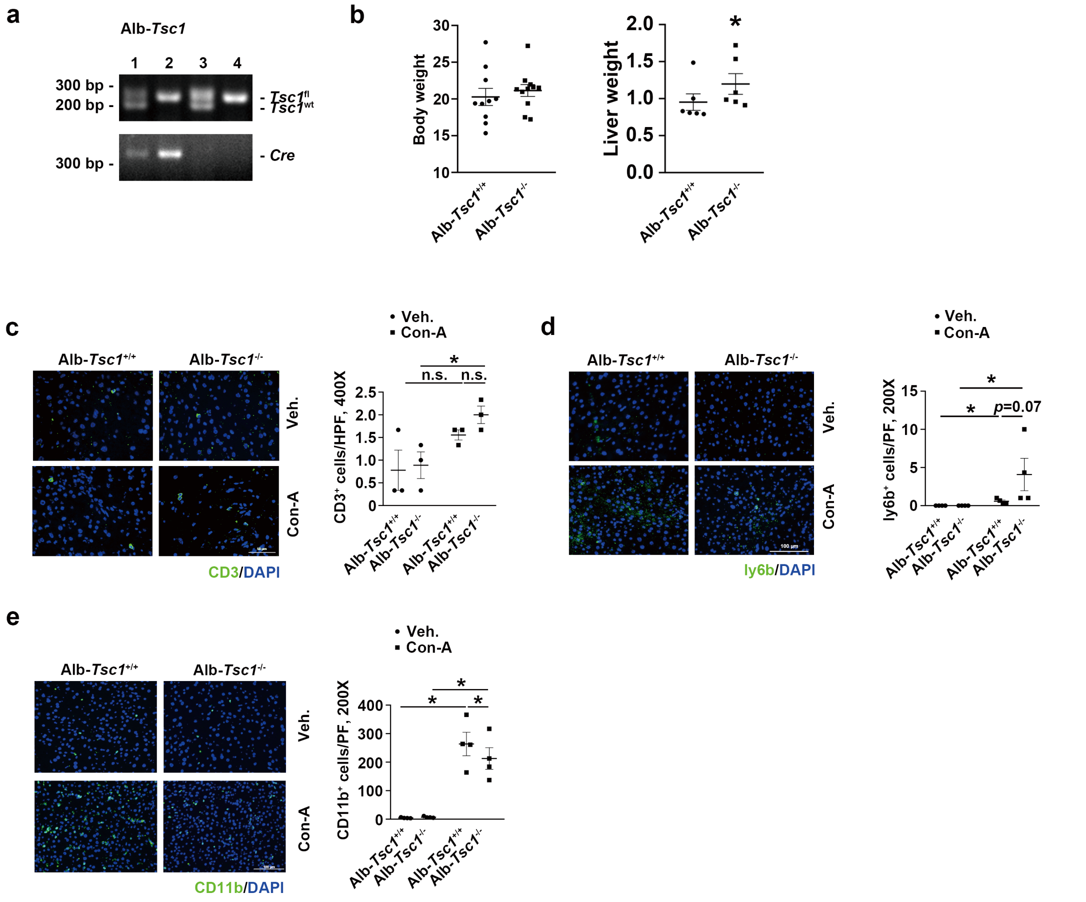

Supplementary Fig. 2 Hepatocyte specific *Tsc1* ablation leads to a diminished inflammatory cells infiltration. a Genotyping the mice by PCR analysis of genomic DNA; numbers (1-4) denote mice with different genotype: 1, Alb-*Tsc1*$^{wt/-}$; 2, Alb-*Tsc1*$^{-/-}$; 3, *Tsc1*$^{fl/wt}$; 4, *Tsc1*$^{fl/fl}$. **b** The liver weight and body weight of Alb-*Tsc1*$^{+/+}$ and Alb-*Tsc1*$^{-/-}$ mice. **c** Left, representative immunofluorescent staining images for CD3. Scale bar = 50 μm. Right, quantitative determination of CD3^{+} cells among groups as indicated, n = 3. **d** Left, representative immunofluorescent staining images for ly6b^{+} cells. Scale bar = 100 μm. Right, quantitative determination of ly6b^{+} cells among groups as

indicated, n = 4. **e** Left, representative immunofluorescent staining images for $CD11b^+$ cells. Scale bar = 100 μm. Right, quantitative determination of $CD11b^+$ cells among groups as indicated, n = 4. * $p<0.05$. Data are expressed as the mean ± SEM. For C-E, significance was determined using the 1-way ANOVA. For B and C, significance was determined using Student 1-tailed t test.

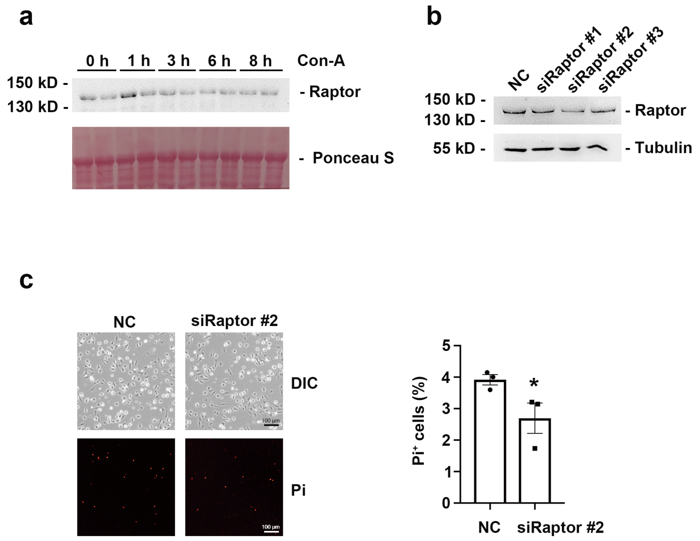

Supplementary Fig. 3 Hepatocyte mTORC1 suppression attenuates KC death. a The expression of Raptor in hepatic tissues. **b**, Primary hepatocytes transfected with siRNAs for Raptor. **c**, Left, representative Pi-staining images for primary KCs with CM from siRaptor #2 transfected hepatocytes for 24 h. Scale bar = 100 μm. Right, quantitative determination of Pi^+ KCs among groups as indicated. * p<0.05, n=3. Data are expressed as the mean ± SEM, significance was determined using Student 1-tailed t test.

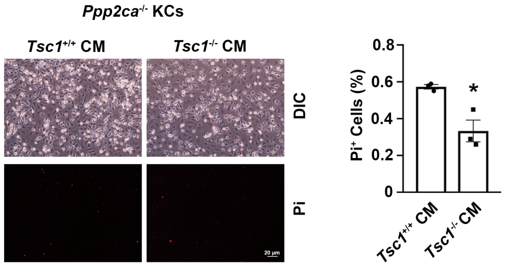

Supplementary Fig. 4 PP2A Cα ablation in KCs represses the *Tsc1*$^{-/-}$ CM-induced KCs death. Left, representative Pi-staining images for *Ppp2Ca*$^{-/-}$ KCs cultured with *Tsc1*$^{+/+}$ or *Tsc1*$^{-/-}$ hepatocytes CM. Scale bar = 20 μm. Right, quantitative determination of Pi^{+} KCs among groups as indicated. * p<0.05, n=3. Data are expressed as the mean ± SEM, significance was determined using Student 1-tailed t test.

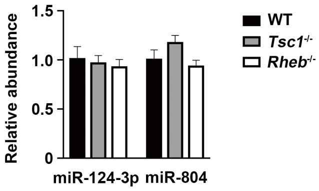

Supplementary Fig. 5 miR-124-3p and miR-804 levels in primary hepatocytes. QRT-PCR assays for miR-124-3p and miR-804 in WT and *Tsc1*$^{-/-}$ primary hepatocytes. Data are expressed as the mean ± SEM, significance was determined using the 1-way ANOVA.

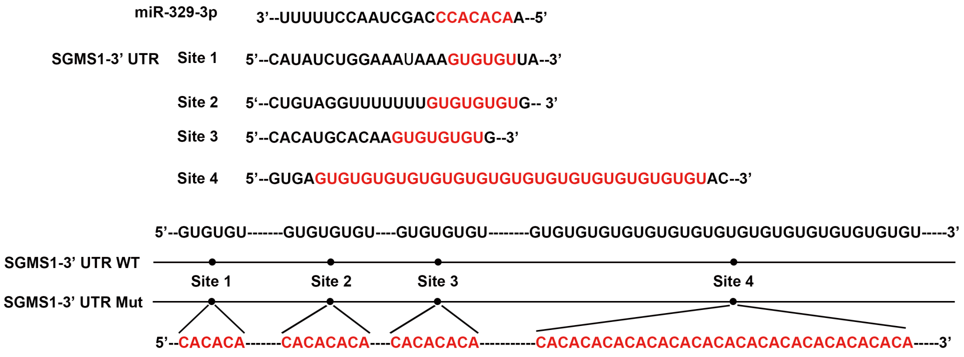

Supplementary Fig. 6 The strategy for the mutation of SGMS1-3'UTR region. SGMS1-3' UTR region and the predicted miR-329-3p binding region (red).

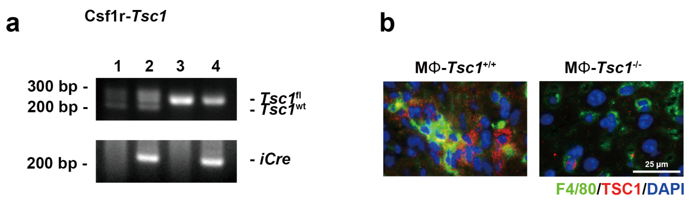

Supplementary Fig. 7 *Tsc1* gene is ablated in KCs. a Genotyping the mice by PCR analysis of genomic DNA; numbers (1-4) denote mice with different genotype: 1, Csf1r-*Tsc1*$^{fl/wt}$; 2, Csf1r -*Tsc1*$^{wt/-}$; 3, Csf1r-*Tsc1*$^{fl/fl}$; 4, Csf1r-*Tsc1*$^{-/-}$. **b** Representative co-immunofluorescent staining images for F4/80 with TSC1. Scale bar = 25 μm.

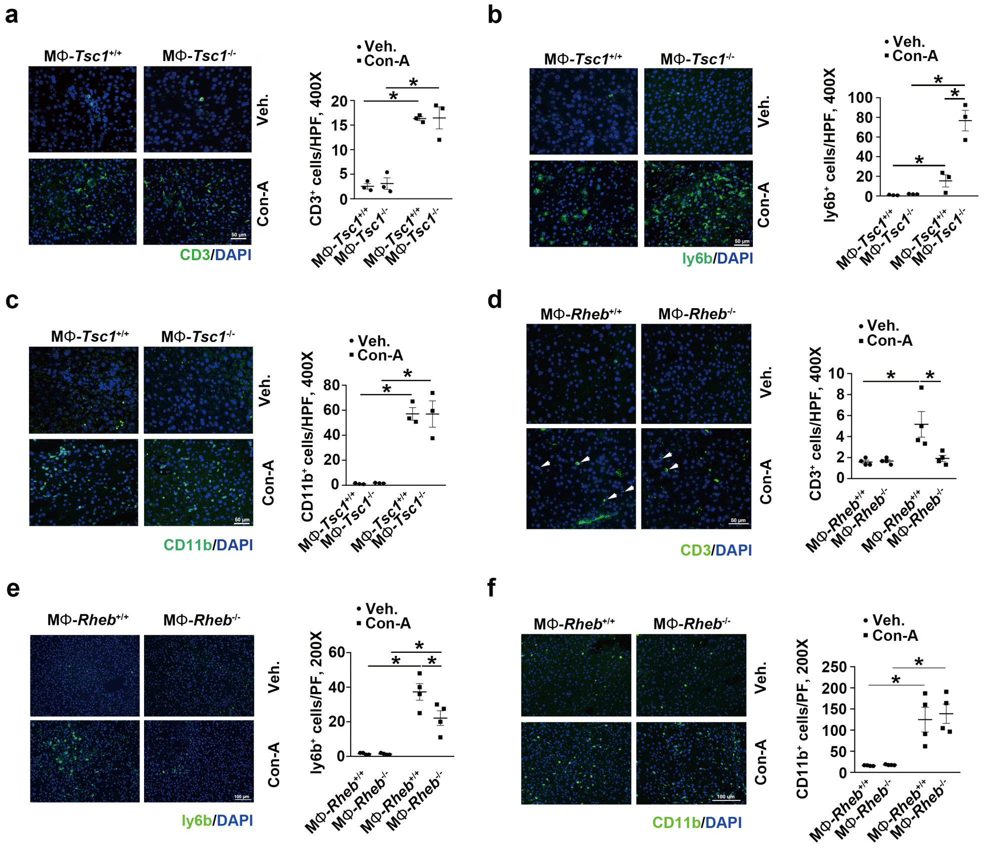

Supplementary Fig. 8 mTORC1 activation in macrophages promotes inflammatory cells infiltration. a Left, representative immunofluorescent staining images for CD3$^+$ cells among groups as indicated. Scale bar = 50 μm. Right, quantitative determination of CD3$^+$ cells among groups as indicated. n = 3. **b** Left, representative immunofluorescent staining images for ly6b$^+$ cells among groups as indicated. Scale bar = 50 μm. Right, quantitative determination of ly6b$^+$ cells among groups as indicated. n = 3. **c** Left, representative immunofluorescent staining images for CD11b$^+$ cells among groups as indicated. Scale bar = 50 μm. Right, quantitative determination of CD11b$^+$ cells among groups as indicated. n = 3. **d** Left, representative

immunofluorescent staining images for $CD3^+$ cells among groups as indicated. $CD3^+$ cells are indicated by white arrows; Scale bar = 50 μm. Right, quantitative determination of $CD3^+$ cells among groups as indicated. n = 4. **e** Left, representative immunofluorescent staining images for $ly6b^+$ cells among groups as indicated. Scale bar = 100 μm. Right, quantitative determination of $ly6b^+$ cells among groups as indicated. n = 4. **f** Left, representative immunofluorescent staining images for $CD11b^+$ cells among groups as indicated. Scale bar = 100 μm. Right, quantitative determination of $CD11b^+$ cells among groups as indicated. n = 4. * $p<0.05$. Data are expressed as the mean ± SEM. For A-F, significance was determined using the 1-way ANOVA.

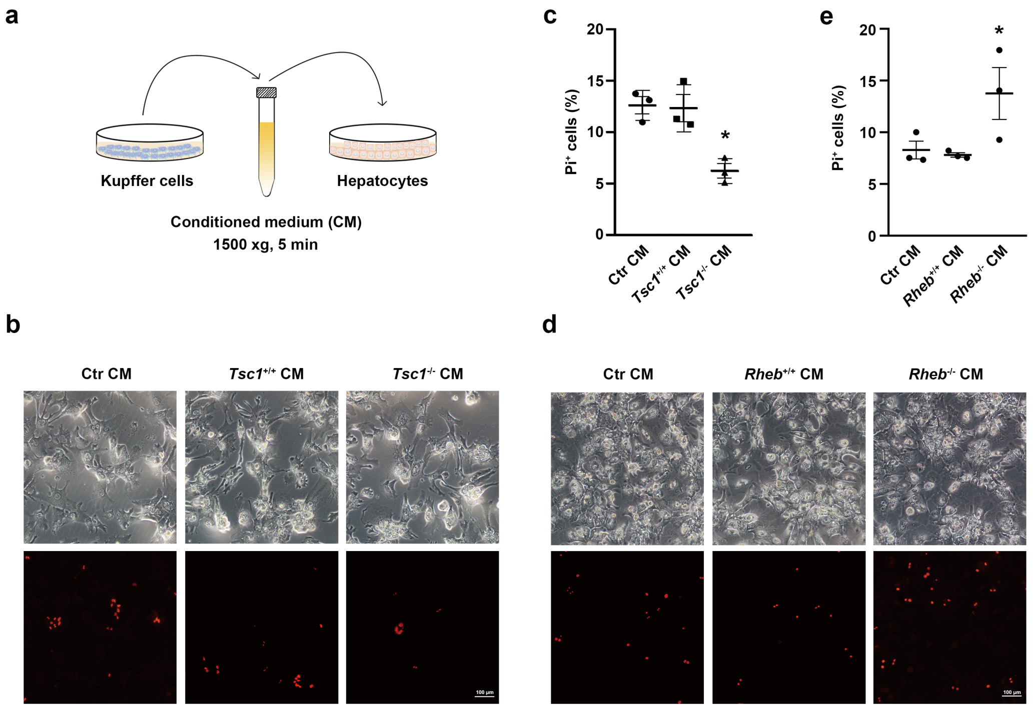

Supplementary Fig. 9 The activation of mTORC1 in KCs protects hepatocytes against to death. a The co-culture system of KCs and hepatocytes. **b** Representative pi-staining images for WT primary hepatocytes cultured with CM for 48 h, which was from WT, *Tsc1*$^{+/+}$ and *Tsc1*$^{-/-}$ primary KCs, respectively. **c** Quantitative determination of Pi^{+} KCs among groups as indicated. n=3. **d** Representative pi-staining images for WT primary hepatocytes cultured with CM for 24 h, which was from WT, *Rheb*$^{+/+}$ and *Rheb*$^{-/-}$ primary KCs, respectively. **e** Quantitative determination of Pi^{+} KCs among groups as indicated. n=3. * p<0.05 versus *Tsc1*$^{+/+}$ CM. Data are expressed as the mean ± SEM. For C and E, significance was determined using the 1-way ANOVA.
